# Long Noncoding RNA LINC00467: Role in Various Human Cancers

**DOI:** 10.3389/fgene.2022.892009

**Published:** 2022-06-01

**Authors:** Di Wu, Rongfei Li, Jingyu Liu, Changcheng Zhou, Ruipeng Jia

**Affiliations:** Department of Urology, Nanjing First Hospital, Nanjing Medical University, Nanjing, China

**Keywords:** LINC00467, microrna sponge, long noncoding RNAs, cancers, biomarker, review

## Abstract

Intricate genetic mutations promote the progression of different cancer types. Long noncoding RNAs (lncRNAs) have been widely demonstrated to participate in the genomic activities of various human cancers. Long intergenic non-coding RNA 467 (LINC00467) is an upregulated lncRNA in diverse diseases, especially in several types of cancers. Functional experiments of LINC00467 revealed that LINC00467 overexpression enhanced cell chemoresistance, proliferation, migration, and invasion in several types of cancers. Moreover, overexpressed LINC00467 was associated with a poor clinical prognosis. The present evidence suggests that LINC00467 may serve as a promising prognostic indicator and become a novel cancer therapeutic target. In this review, we introduce the biologic functions of lncRNAs and describe the molecular mechanism and clinical significance of LINC00467 in detail.

## 1 Introduction

Cancer, with high morbidity and mortality, has long been known as a primary life-threatening disease worldwide and imposes a huge economic and social burden on every country. (Sung et al., 2021) In recent years, the life expectancy of patients with cancer has greatly increased owing to the earlier detection and appropriate therapies. However, several cancer complications such as tumor metastasis, drug resistance, and cancer recurrence hinder the management of this disease and contribute to the high mortality associated with it. (Barik et al., 2021) Although traditionally most research about these processes has focused on the role of protein-coding RNAs (Slack and Chinnaiyan, 2019), recent efforts have focused on non-coding RNAs, which are now known to be regulatory molecules that mediate cellular processes and play an important role in tumorigenesis and cancer development. (Anastasiadou et al., 2018)

Long non-coding RNAs (lncRNAs) are RNA molecules longer than 200 nucleotides and comprise the main category of non-coding RNAs (Palazzo and Koonin, 2020) According to the latest release of the database NONCODE, the number of human lncRNAs is approximately 170,000, which is much larger than that of protein-coding RNAs. (Derrien et al., 2012; Zhao et al., 2021) Most lncRNAs are transcribed by RNA polymerase II, and then are capped at the 5′ end, ploy A tail added at the 3′ end, and edited to form biologically functional lncRNAs. (Statello et al., 2021) According to classification based on function, lncRNAs can be divided into activating ncRNAs, competing endogenous RNA (ceRNAs), and precursors for shorter functional RNA. (St Laurent et al., 2015) Most lncRNAs act as ceRNAs. The “competitive endogenous RNA hypothesis,” which was put forward by Salmena et al., in 2011, proposes that lncRNAs regulate mRNA expression by targeting miRNAs through homologous miRNA response elements (MREs). By binding to MREs present in miRNAs, lncRNAs inactive them, a process known as the “molecular sponge effect”, suppressing the inhibitory effect of the targeted miRNA on mRNA expression. (Salmena et al., 2011) Numerous posterior studies have confirmed the lncRNA-miRNA-mRNA axis established by this hypothesis, as well as the role of this axis in tumor development.

Long intergenic non-coding RNA (lincRNA) is defined as ‘lncRNA not overlapping a protein-coding transcript’. (Ransohoff et al., 2018) LINC00467, a newly defined lincRNA, is located at Chromosome 1: 211,382,736-211,444,093 (GRCh38/hg38) with the length of 61,358 bases (www.genecards.org). Moreover, it encodes 28 different transcripts (www.ensembl.org). In addition to being upregulated in normal human testis and kidney tissues (Fagerberg et al., 2014), recent studies have confirmed that LINC00467 is overexpressed in various types of human tumor tissues and plays an important role in regulating gene transcription, tumor initiation, and progression.

In this review, we comprehensively summarize the different functional roles of the newly found LINC00467 in the progression of various cancers. LINC00467 mainly functions as a ceRNA and leads to the overexpression of relevant genes by sponging the corresponding miRNAs. Additionally, we present the clinical significance of LINC00467 in several human cancers and discuss its potential clinical application as an early diagnostic and prognostic biomarker and novel therapeutic target.

## 2 Function of lncRNAs

**FIGURE 1:**
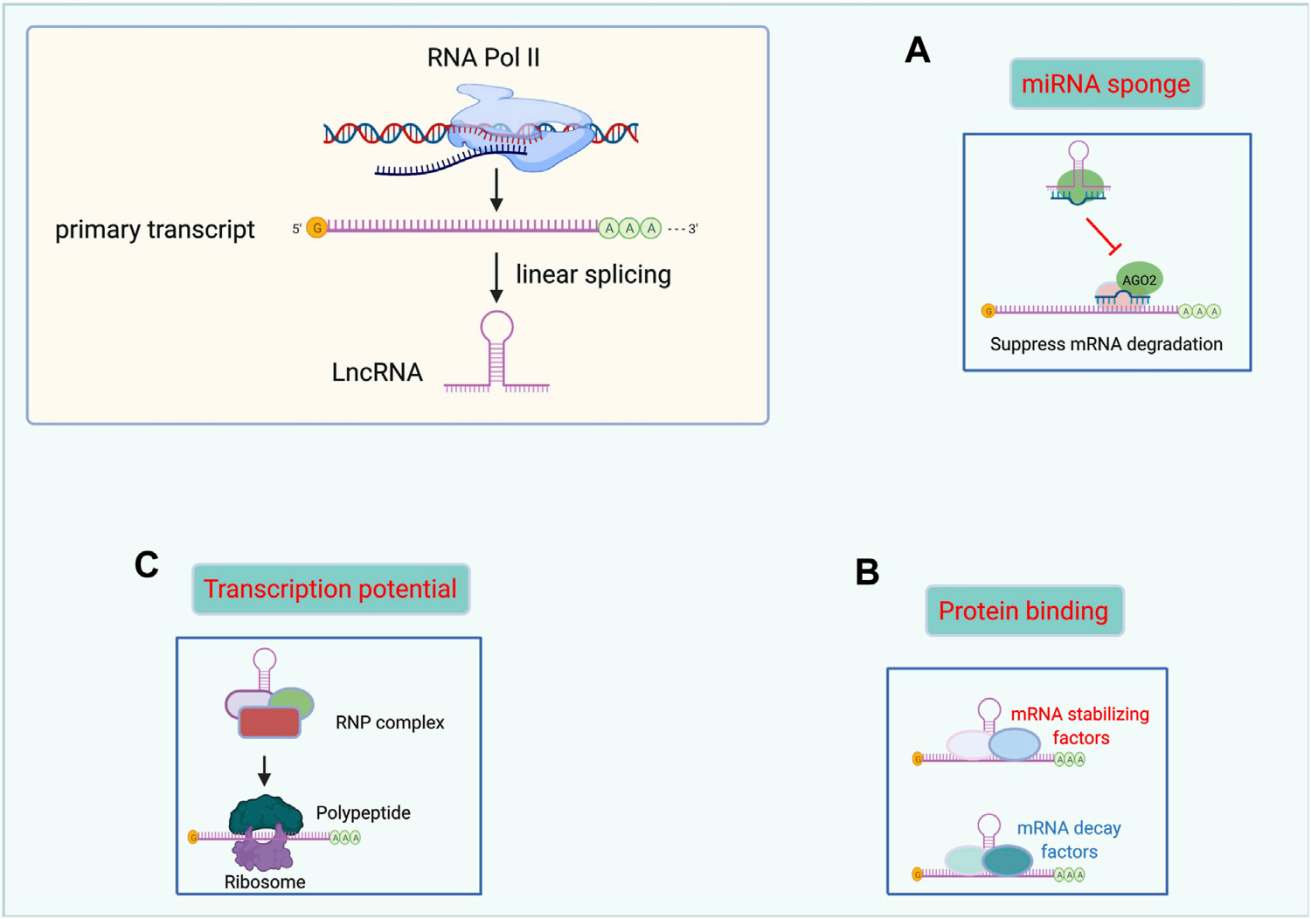
Functional mechanisms of lncRNAs. lncRNAs can act as miRNAs sponges **(A)**, binding with proteins **(B)**, and templates for polypeptide translation **(C)**. Abbreviations: lncRNA, Long non-coding RNA. miRNAs, microRNAs. RNP complex, ribonucleoprotein complex.

Proteins are universally acknowledged to be the basis of life. However, “non-protein-coding” is not the same as being useless. Mounting studies have demonstrated the indispensable and promising role of lncRNAs in many diseases. Herein, we summarized the main functional mechanisms of lncRNAs in the following paragraphs. Moreover, we made a figure to illustrate the function of lncRNAs more graphically. (Figure 1).

### 2.1 Acting as miRNA Sponges

The sponging of miRNAs is one of the most studied mechanisms of action of lncRNAs. As we know, some miRNAs could effectively bind to certain mRNAs in the presence of MRE, a “seed sequence” containing two to eight nucleotides. Interestingly, many lncRNAs, including lincRNAs, pseudogene transcripts, and circular RNAs share the same MRE with certain miRNAs. Then, a series of miRNAs were bound with these ceRNAs rather than mRNAs. As a result, the inhibitory effect of targeted binding of miRNA to mRNA was attenuated by lncRNAs. Through this regulatory mechanism, lncRNAs play an important role in the biological progression of many cancers. (Kazimierczyk et al., 2020) In prostate cancer, LINC01679 has been shown to inhibit cell proliferation, invasion, and tumor growth, and promote cell apoptosis *in vivo* and *in vitro* by competitively binding to miR-3150a-3p as well as by regulating the downstream gene *SLC17A9*. (Mi et al., 2021) What’s more, recent evidence indicates that one lncRNAs may have several MREs to sponge different miRNAs. In acute myeloid leukemia (AML), the lncRNA MIR17HG sponges miR-21 to further promote homoharringtonine-induced AML cell apoptosis. (Yan et al., 2022) Besides MIR17HG also inhibits non-small cell lung cancer *via* functioning as a ceRNA of miR-142-3p. (Wei et al., 2020) Through this regulatory mode, lncRNAs are widely involved in the course of various diseases, including thyroid cancer (Guo et al., 2021), renal fibrosis (Tang et al., 2021), atherosclerotic plaques (Ann et al., 2021), among others.

### 2.2 Interaction With Proteins

Due to their large size of hundreds, even thousands of nucleotides, lncRNAs have been shown to function as guides, signals, decoys, and scaffolds for many different proteins. (Kazimierczyk et al., 2020) Mounting evidence notes that many lncRNAs can directly bind or form complexes with some key proteins to play diverse regulatory roles including transcription and splicing, as well as gene expression. (Wu et al., 2018) A study on gallbladder cancer revealed that the lncRNA RP11-147L13.8 serves as a tumor-suppressing gene by interacting with c-Jun protein via binding to its bZIP domain (a DNA-binding and dimerization domain) and suppressing c-Jun-ser73 phosphorylation. (Zheng et al., 2021) Interestingly, another study on gallbladder cancer found that HuR protein specifically binds with lncRNAs-HGBC within the 1759–1906 nt-long region and enhances the stabilization of lncRNAs-HGBC (Hu et al., 2019) Moreover, the lncRNA AK137033 was found to interact with mRNA Sfrp2 and HuR, forming the AK137033-Sfrp2-HuR complex, which contributes to the inhibition of cardiac fibrosis. (Hao et al., 2019)

### 2.3 Encoding Polypeptides

LncRNA was named as ‘non-coding RNA’ for lack of an open reading frame. However, recent research has shown that some lncRNAs can encode polypeptides that are usually shorter than 100 amino acids and widely involved in tumorigenesis, metabolism, inflammation, and signal transduction pathways. (Ye et al., 2020) Pang et al (Pang et al., 2020) reported that the lncRNA Linc00998 encodes the micro peptide SMIM30, which facilitated HCC cell proliferation and migration and was associated with a poorer survival rate in HCC patients. Similarly, Linc00617 encodes BNLN, which maintains the stabilization of islet *ß* cells and raises insulin secretion in islets from humans. (Li M. et al., 2021) In addition, Niu et al (Niu et al., 2020) demonstrated that the lncRNA MIR155HG encodes another micro peptide, named miPEP155, which functions as an important regulator of antigen presentation as well as a suppressor of inflammatory diseases. This new functional mechanism of lncRNAs may become a new hotspot for future research on cancer biomarkers and drug development.

## 3 Clinical Significance of LINC00467 in Various Human Cancers

Various studies have investigated the expression of LINC00467 in different tumor tissues and corresponding controls (mostly adjacent non-malignant tissues). All studies (except that by Cai et al. (2019)) indicated that the expression of LINC00467 was upregulated in a variety of cancer tissues compared to the control group. In addition, numerous studies of different tumor types have assessed the prognosis of patients with high or low LINC00467 expression level and its association with prognostic features such as TNM (Tumor, Node, Metastasis) staging, cell differentiation, disease-free survival, and overall survival. Patients with high expression of LINC00467 tend to have advanced TNM stage, poorer cell differentiation, and shorter disease-free survival and overall survival. The details of relevant studies have been summarized in Table 1.

**TABLE 1:** Clinical significance of LINC00467 in various human tumors. (ANCTs: adjacent non-cancerous tissues).

Cancer Type	Sample Size	Expression (tumor Vs. normal)	Clinicopathological Features	Reference
Non-small cell lung cancer (NSCLC)	91 pairs of NSCLC tissues and ANCTs	Upregulation	Advanced clinical stages, shorter overall survival and disease-free survival	Xue et al. (2021)
	GEPIA database	Not studied	Advanced clinical stages	Zhu et al. (2020)
Lung adenocarcinoma (LUAD)	60 pairs of LUAD tissues and ANCTs	Upregulation	Shorter overall survival	Wang et al. (2019)
	33 pairs of LAD tissues and ANCTs	Upregulation	Not studied	Ding et al. (2019)
Hepatocellular carcinoma (HCC)	65 HCC tissues and 31 ANCTs	Downregulation	Advanced clinical stages	Cai et al. (2019)
	56 pairs of HCC tissues and ANCTs	Upregulation	Shorter overall survival	Wang et al. (2020)
	20 pairs of HCC tissues and ANCTs	Upregulation	Not studied	Zheng et al. (2020)
	GEPIA database	Upregulation	Advanced clinical stages, poorer cell differentiation, shorter overall survival and disease-free survival	Jiang et al. (2020)
	GEPIA database	Upregulation	Contributed to Axitinib resistance	Li et al. (2021b)
Cervical Cancer Cells	54 pairs of Cervical Cancer tissues and ANCTs	Upregulation	Advanced clinical stages, shorter overall survival	Li et al. (2019)
Osteosarcoma (OS)	44 pairs of OS tissues and ANCTs	Upregulation	Advanced clinical stages, shorter overall survival	Ma et al. (2020)
	36 pairs of OS tissues and ANCTs	Upregulation	Advanced clinical stages, shorter overall survival	Yan et al. (2021)
Glioma	30 matched glioma tissues and normal tissues	Upregulation	Shorter overall survival	Jiang and Liu, (2020)
	TCGA STAD database	Upregulation	Not studied	Zhang et al. (2020)
Glioblastoma	30 matched Glioblastoma tissues and normal tissues	Upregulation	Advanced clinical stages	Liang and Tang, (2020)
Colorectal cancer (CRC)	31 pairs of CRC tissues and ANCTs	Upregulation	Shorter overall survival and disease-free survival	Bai et al. (2020)
	45 matched CRC tissues and normal tissues	Upregulation	Not studied	He et al. (2019)
	GEO dataset	Upregulation	Shorter overall survival and disease-free survival	Ge et al. (2021)
	50 CRC tissues and 10 normal tissues	Upregulation	Shorter overall survival	Li et al. (2020)
Testicular germ cell tumors (TGCT)	14 TGCT tissues and 9 ANCTs	Upregulation	Not studied	Bo et al. (2021)
Prostate cancer (PC)	60 pairs of PC tissues and ANCTs	Upregulation	Shorter overall survival	Jiang et al. (2021)
Breast Cancer	70 pairs of Breast Cancer tissues and ANCTs	Upregulation	Not studied	Zhang et al. (2021)
Esophageal squamous cell carcinoma (ESCC)	44 pairs of ESCC tissues and ANCTs	Upregulation	Not studied	Liu et al. (2021)
Head and neck squamous cell carcinoma (HNSCC)	45 HNSCC tissues and 20 ANCTs	Upregulation	Advanced clinical stages, poorer cell differentiation	Liang et al. (2021)
Gastric cancer (GC)	31 pairs of GC tissues and ANCTs	Upregulation	Not studied	Xu et al. (2021)
	TCGA STAD database	Upregulation	Shorter overall survival	Deng et al. (2021)
Bladder Cancer (BCa)	6 pairs BCa tissues and ANCTs	Upregulation	Not studied	Xiao et al. (2021)
Acute myeloid leukemia (AML)	134 BM specimens from AML patients and 40 healthy controls	Upregulation	Not studied	Lu et al. (2021)

## 4 Molecular Mechanism of LINC00467 in Diverse Human Cancers

**FIGURE 2:**
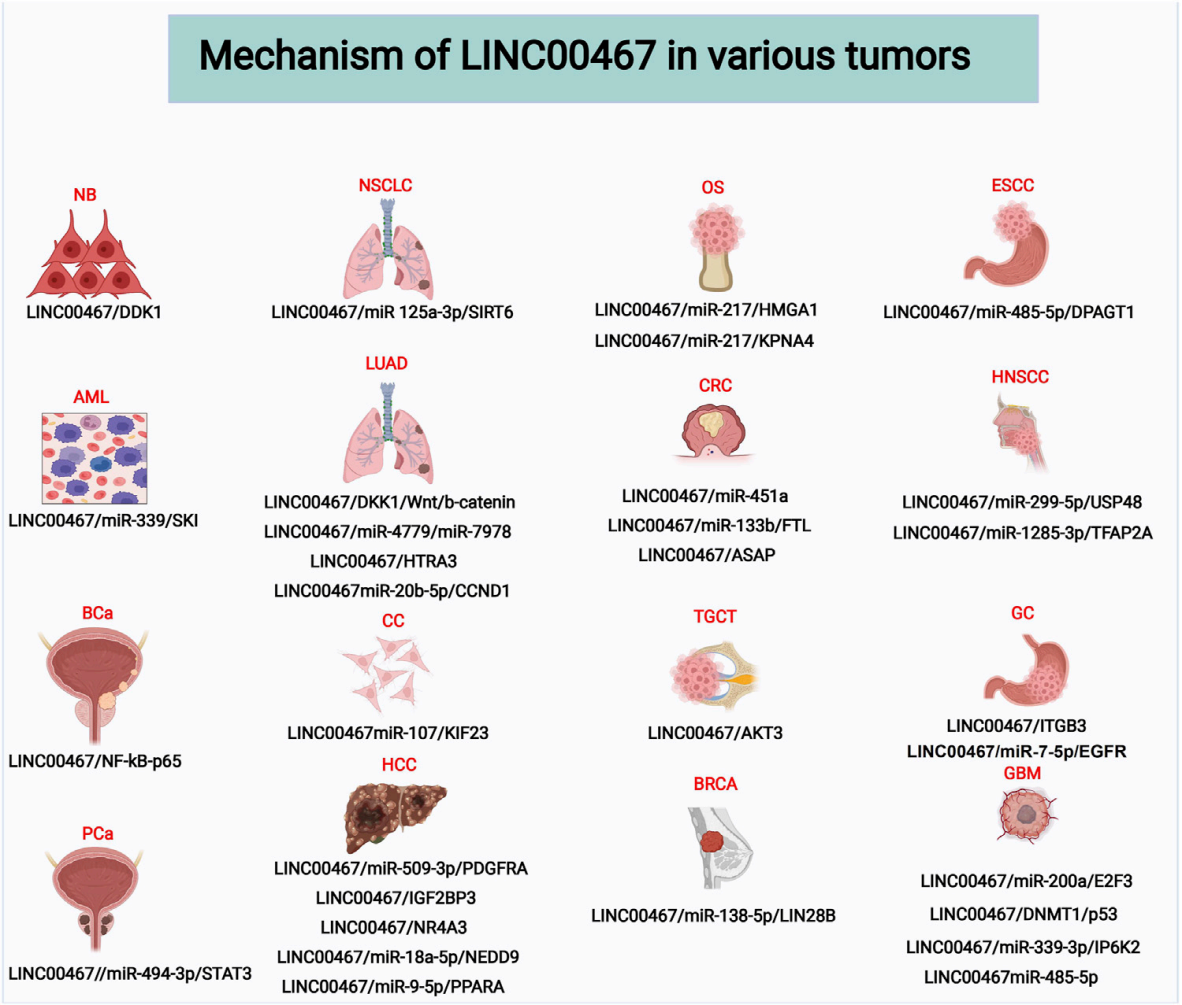
Molecular mechanisms of LINC00467 in various tumors. Abbreviations: NB, Neuroblastoma; NSCLC, Non-small cell lung cancer; OS, Osteosarcoma; ESCC, Esophageal squamous cell carcinoma; AML, Acute Myeloid Leukemia; LUAD, Lung adenocarcinoma; CRC, Colorectal cancer; HNSCC, Head and neck squamous cell carcinoma; BCa, Bladder Cancer; CC, Cervical cancer; TGCT, Testicular germ cell tumor; GC, Gastric cancer; PCa, Prostate cancer; HCC, Hepatocellular carcinoma; BRCA, Breast Cancer; GBM, Glioblastoma.

To date, dozens of studies have reported the effects of LINC00467 in various human cancer cell lines, including its impact on tumor cell proliferation, invasion, migration, and apoptosis (Table 2). In the following sections, we elaborate on the functional roles and molecular mechanism of LINC00467 in various cancers. Additionally, another figure was made to illustrate these molecular mechanisms of LINC00467. (Figure 2)

**TABLE 2:** Functional molecular mechanisms of LINC00467 in various cancers.

Tumor Types	Expression	Function Role	Molecular Mechanism	References
Neuroblastoma	Not Studied	neuroblastoma cell survival	Inducing DKK1 expression	Atmadibrata et al. (2014)
Acute myeloid leukemia (AML)	Upregulation	progression	LINC00467/miR-339/SKI axis	Lu et al. (2021)
Bladder Cancer (BCa)	Upregulation	Proliferation, invasion, and progression	Interacting with NF-kB-p65	Xiao et al. (2021)
Prostate cancer (PC)	Upregulation	proliferation, migration, and invasion	LINC00467/miR-494-3p/STAT3 axis	Jiang et al. (2021)
Non-small cell lung cancer (NSCLC)	Upregulation	proliferation, migration, invasion, EMT, inhibit apoptosis	LINC00467/miR-125a-3p/SIRT6 axis	Xue et al. (2021)
		progression, proliferation, and metastasis	Enhancing the Akt signaling pathway	Zhu et al. (2020)
Lung adenocarcinoma (LUAD)	Upregulation	proliferation, migration	Silencing DKK1 to activate the Wnt/b-catenin signaling pathway	Yang et al. (2019)
		proliferation, stemness, inhibit apoptosis	Sponging miR-4779 and miR-7978	Chang and Yang, (2019)
		proliferation, migration, and invasion, inhibit apoptosis	Repressing HTRA3	Wang et al. (2019)
		proliferation and cell cycle progression	LINC00467/miR-20b-5p/CCND1 axis	Ding et al. (2019)
		Not studied	LINC00467/hsa-miR-1225-5p, hsa-miR-575/BARX2, BCL9, KCNK1, KIAA1324, TMEM182	Wang et al. (2021)
Cervical cancer	Upregulation	proliferation, migration, invasion, and EMT	LINC00467/miR-107/KIF23 axis	Li et al. (2019)
Hepatocellular carcinoma (HCC)	Upregulation	proliferation, invasion, repressed apoptosis	LINC00467/miR-509-3p/PDGFRA axis	Li et al. (2021b)
		proliferation metastasis, inhibit apoptosis	Binding with IGF2BP3	Jiang et al. (2020)
		proliferation, progression, migration, inhibit apoptosis	Inhibiting NR4A3	Wang et al. (2020)
		proliferation, invasion, inhibit apoptosis	LINC00467/miR-18a-5p/NEDD9 axis	Zheng et al. (2020)
	Upregulation	impeding cell viability, proliferation, migration, and invasion	LINC00467/miR-9-5p/PPARA axis	Cai et al. (2019)
Osteosarcoma (OS)	Upregulation	proliferation, migration, invasion, inhibit apoptosis	LINC00467/miR-217/HMGA1 axis	Ma et al. (2020)
	Upregulation	proliferation, migration, invasion, and EMT	LINC00467/miR-217/KPNA4 axis	Yan et al. (2021)
Colorectal cancer (CRC)	Upregulation	proliferation, invasion	Not studied	He et al. (2019)
	Upregulation	proliferation, metastasis, inhibit apoptosis	Sponging miR-451a	Bai et al. (2020)
	Upregulation	Chemoresistance to 5-FU, metastasis	LINC00467/miR-133b/FTL axis	Li et al. (2020)
	Upregulation	proliferation	Encoding ASAP	Ge et al. (2021)
Testicular germ cell tumors (TGCT)	Upregulation	migration, invasion, inhibit immune cells activation and infiltration	Activating AKT3	Bo et al. (2021)
Breast Cancer	Upregulation	proliferation, migration, invasion, and EMT	LINC00467/miR-138-5p/LIN28B axis	Zhang et al. (2021)
Esophageal squamous cell carcinoma (ESCC)	Upregulation	proliferation, invasion, inhibit apoptosis	LINC00467/miR-485-5p/DPAGT1 axis	Liu et al. (2021)
Head and neck squamous cell carcinoma (HNSCC)	Upregulation	proliferation, migration, EMT, invasion	LINC00467/miR-299-5p/USP48 axis	Chen and Ding, (2020)
	Upregulation	invasion and inhibit apoptosis	LINC00467/miR-1285-3p/TFAP2A axis	Liang et al. (2021)
Gastric cancer (GC)	Upregulation	invasion, proliferation, inhibit apoptosis	Enhancing ITGB3 level	Xu et al. (2021)
	Upregulation	proliferation, migration, and invasion	LINC00467/miR-7-5p/EGFR axis	Deng et al. (2021)
Glioma	Upregulation	viability, migration, and invasion	LINC00467/miR-200a/E2F3 axis	Gao et al. (2020)
	Upregulation	proliferation, invasion, cell cycle, inhibit apoptosis	LINC00467/DNMT1/Ip53 axis	Zhang et al. (2020)
	Upregulation	proliferation, inhibit apoptosis	LINC00467/miR-339-3p/IP6K2 axis	Liang and Tang, (2020)
	Upregulation	proliferation, invasion, inhibit apoptosis	Sponging miR-485-5p	Jiang and Liu, (2020)

### 4.1 Function as a ceRNA

Based on the currently published research, the most reported mechanism is that LINC00467 functions as a ceRNA of a series of miRNAs. LINC00467 forms the regulatory axis of LINC00467-miRNA-mRNA by regulating downstream targeted coding genes through competitive inhibition with some certain miRNA. Through this regulatory pattern, LINC00467 could participate in the initiation and development of cancers.

#### 4.1.1 Acute Myeloid Leukemia

In the United States, AML accounts for approximately one-third of all leukemias diagnosed and 1.2% of all annual new cancer diagnoses (Pelcovits and Niroula, 2020) The role of LINC00467 in AML pathogenesis was recently researched by Lu et al. (2021) The results of this study showed that LINC00467 expression was associated with the malignant phenotypes of AML cells and that it acted as a ceRNA of miR-339. SKI is a gene regulated by miR-339 that has been reported to promote AML development. Further investigation of the underlying mechanism verified that treatment with a miR-339 inhibitor largely diminished the influence of LINC00467 knockdown on AML cells. These results suggest that targeting the LINC00467/miR-339/SKI axis could be a suitable approach for the treatment of AML.

#### 4.1.2 Non-Small Cell Lung Cancer

NSCLC accounts for nearly 85% of lung cancers (Herbst et al., 2008; Yin et al., 2018) and is known to be the most common cause of cancer-related death worldwide. (Siegel et al., 2018) Most of the patients diagnosed with NSCLC are already in advanced stages for which effective treatment options are still lacking (Hayden and Ghosh, 2008; Saha et al., 2020) LINC00467 regulates ERK1/2 signaling in NSCLC. Inhibiting SIRT6 and blocking the ERK1/2 signaling pathway lead to the upregulation of miR-125a-3p and the downregulation of LINC00467 in NSCLC cells. Additionally, LINC00467 upregulation or miR-125a-3p downregulation exacerbated the malignant phenotypes and DDP resistance in NSCLC cells. (Xue et al., 2021).

#### 4.1.3 Lung Adenocarcinoma

LUAD is the commonest histological subtype of NSCLC. Early diagnosis of LUAD is particularly difficult and it is prone to metastasize, as a result of which it is associated with a poor prognosis. (Yang et al., 2019) LINC00467 was found to be upregulated in human LUAD tumor tissues and its expression level was found to correlate with the clinical severity of LUAD. Functional *in vitro* experiments indicated that LINC00467 promoted LUAD cell proliferation, migration, and invasion and suppressed apoptosis. Furthermore, some miRNAs sponged by LINC00467 and their downstream targets, which play a role in LUAD development, have been reported. For instance, a study found that LINC00467 can suppress apoptosis and promote proliferation and stemness of LUAD cells by sponging cytoplasmic miR-4779 or miR-7978. (Chang and Yang, 2019) Additionally, Ding et al. (2019) reported that LINC00467 promotes proliferation in LUAD cells by upregulating the expression level of CCND1, a primary regulator of the cell cycle, via sponging miR-20b-5p.

Finally, Wang et al. reported that LINC00467 DNA copy number amplification contributed to its overexpression and was positively associated with distant metastasis, immune infiltration, and poor overall survival in LUAD. In addition, LINC00467 overexpression partly originated from its DNA demethylation. Based on microarray analysis and bioinformatics, this study revealed a comprehensive miRNAs-mRNAs network that included two microRNAs (hsa-miR-1225-5p, hsa-miR-575) and five mRNAs (BARX2, BCL9, KCNK1, KIAA1324, TMEM182) specific to LINC00467 in LUAD. Through bioinformatic analysis, two potential prognostic biomarkers (BARX2 and BCL9) for LUAD were revealed. (Wang et al., 2021) Based on the results of these reports, LINC00467 stands as a potential diagnostic and prognostic biomarker, as well as a suitable therapeutic target for LUAD.

#### 4.1.4 Cervical Cancer

In the developing world, cervical cancer remains the second most common cancer among women, thereby placing a heavy economic burden on society.(Hillmann et al., 2013; Siva et al., 2015) It has been reported that cytoplasmic LINC00467 plays an important role in the progression of cervical cancer by competitively sponging miR-107 to downregulate the expression of the downstream gene KIF23. LINC00467 silencing or miR-107 overexpression inhibited cervical cancer cell proliferation, migration, invasion, epithelial-mesenchymal transition (EMT), and tumorigenic ability *in vivo*, further suggesting that targeting LINC00467 may be a novel therapeutic strategy for patients with cervical cancer. (Li et al., 2019).

#### 4.1.5 Hepatocellular Carcinoma

HCC is a prevalent human malignancy with high morbidity and mortality worldwide.(Maluccio and Covey, 2012; Laursen, 2014) Exploring novel therapeutic targets for HCC remains a pressing challenge nowadays (Dhar et al., 2018; Andrade et al., 2019) LINC00467 expression is upregulated in HCC cells and is related to HCC progression. Additionally, miR-509-3p and platelet-derived growth factor receptor alpha (PDGFRA), which are targets of LINC00467, promoted proliferation and invasion, repressed apoptosis, and ameliorated the resistance to axitinib, the commonly-used drug for HCC (Chan et al., 2017; Li W. et al., 2021) Moreover, Zheng et al. described that silencing LINC00467 could modulate HCC cell growth and development by targeting the miR-18a-5p/NEDD9 axis. (Zheng et al., 2020) Interestingly, one study reported that the expression of LINC00467 was significantly decreased in HCC tissues, especially in metastatic HCC tissues, which is in contrast to the results of other relevant studies on LINC00467 in HCC. Furthermore, it was reported that LINC00467 exerted a tumor suppressor role by reducing cell viability, proliferation, migration, and invasion by regulating the miR-9-5a/PPARA signaling axis in HCC. (Cai et al., 2019).

#### 4.1.6 Osteosarcoma

OS, which mainly occurs in children and adolescents, has a poor prognosis and low survival rate due to its rapid progression and metastasis ability.(Wycislo and Fan, 2015; Marko et al., 2016) Ma et al. reported that LINC00467 expression was increased in OS cell lines, and that overexpression of LINC00467 increased cell proliferation, migration, and invasion, and decreased cell apoptosis by upregulating the expression of high mobility group A1 (HMGA1) by targeting miR-127 in OS. (Ma et al., 2020) Additionally, it was reported that LINC00467-mediated sponging of miR-127 also regulated the expression of karyopherin subunit *α* 4 (KPNA4), contributing to OS progression. (Yan et al., 2021).

#### 4.1.7 Colorectal Cancer

CRC is the second leading cause of cancer-related mortality worldwide, partly due to the lack of effective treatments.(Walsh and Terdiman, 2003; Sunkara and Hébert, 2015; Sung et al., 2021) LINC00467 expression level has been reported to be significantly increased in CRC tissues and cell lines. Silencing its expression resulted in the inhibition of CRC cell proliferation, invasion, and EMT. (He et al., 2019) Another study using data from 31 CRC patients further elucidated that LINC00467 upregulation was closely associated with metastasis and TNM stage in CRC. Moreover, the authors reported that overexpression of miR-451a reduced the level of LINC00467 in CRC cells. (Bai et al., 2020) Additionally, LINC00467 has also been suggested to play other biological roles in CRC cells, including mediating chemoresistance to 5-FU and metastasis, through the miR-133b/ferritin light chain (FTL) axis. (Li et al., 2020).

#### 4.1.8 Prostate Carcinoma

PC, which is a heterogeneous disease, is the most common non-skin male malignancy in the Western world. (Haffner et al., 2021) LINC00467 expression is upregulated in PC tissues and cells. Meanwhile, LINC00467 knockdown reduced PC cell proliferation, migration, and invasion via regulation of the miR-494-3p/STAT3 axis. Additionally, it has also been shown that downregulation of LINC00467 inhibited M2 macrophage polarization and suppressed the migration and invasion of PC cells by activating the STAT3 pathway. These results suggest that inhibiting LINC00467 may be an effective therapeutic strategy for patients with PC. (Jiang et al., 2021).

#### 4.1.9 Breast Cancer

Breast cancer is the most common type of malignancy among women and is associated with a high number of cancer-related deaths worldwide. (Jemal et al., 2011) Zhang et al. reported that silencing LINC00467 hindered the proliferation, migration, invasion, and EMT of breast cancer cell lines *in vitro*, whereas overexpression of LINC00467 predicted poor overall survival. Additional experiments have revealed that LINC00467 sponges miR-138-5p while directly interacting with LIN28B, a significant oncogene in breast cancer, to elevate its protein level. (Zhang et al., 2021).

#### 4.1.10 Esophageal Squamous Cell Carcinoma

ESCC, an important subtype of esophageal carcinoma, is the most common primary gastrointestinal malignant tumor. It has a relatively low 5-years survival rate and a high incidence, with more than 400,000 newly-diagnosed cases annually worldwide (Luo et al., 2019; Sugimura et al., 2019) LINC00467 expression is upregulated in ESCC tissues and cell lines, and high LINC00467 expression is correlated with the promotion of cell proliferation and inhibition of cell apoptosis. More importantly, LINC00467 facilitated ESCC progression by sponging miR-485-5p and modulating DPAGT1 levels. (Liu et al., 2021).

#### 4.1.11 Head and Neck Squamous Cell Carcinoma

HNSCC, which mainly affects the oral cavity, pharynx, and larynx, is among one the ten malignancies with more than 600,000 new cases worldwide annually (Siegel et al., 2017; Xu et al., 2020) The notable upregulation of LINC00467 in HNSCC promotes cell growth, cell migration, and EMT by sponging miR-299-5p, thereby upregulating the expression of the oncogene tumor ubiquitin specific protease-48 (USP48), ultimately leading to HNSCC progression. (Chen and Ding, 2020) Additionally, Liang et al. reported that LINC00467 over-expression in HNSCC patients promoted cell invasion and inhibited apoptosis by sponging miR-1285-3p and modulating the expression level of TFAP2A, a downstream target of miR-1285-3p. (Liang et al., 2021).

#### 4.1.12 Gastric Cancer

GC is an aggressive malignant tumor with a high mortality rate that is often diagnosed at an advanced stage. (Abdi et al., 2020) A study found that LINC00467 knockdown inhibited proliferation, migration, and invasion of GC cells. Furthermore, researchers revealed that LINC00467 promotes GC progression by directly regulating miR-7-5p/EGFR (epidermal growth factor receptor) axis. (Deng et al., 2021).

#### 4.1.13 Glioma

Gliomas are tumors derived from the neuroepithelial ectoderm that stand among the most prevalent intracranial tumors, accounting for approximately 40% of all brain tumors. (Chen et al., 2017) Gao et al. demonstrated that high LINC00467 expression is a distinctive feature of glioma cell lines compared with normal cell lines. Moreover, LINC00467 was shown to promote the viability, migration, and invasion of glioma cells, and its expression level was negatively correlated with that of miRNA-200a, but positively correlated with E2F3 expression. LINC00467 accelerated the progression of gliomas by regulating the miR-200a/E2F3 axis. (Gao et al., 2020) Zhang et al. showed that increased LINC00467 expression in glioma cell lines promoted tumor proliferation, invasion, and cell cycle progression, but inhibited cell apoptosis by binding to DNMT1 to inhibit the expression of the tumor suppressor P53. (Zhang et al., 2020) In addition, another study found that LINC00467 exerted its biological function, including accelerating the proliferation and invasion abilities of glioma cells, as well as attenuating apoptosis, by sponging miRNA-485-5p (Jiang and Liu, 2020).

Glioblastoma, which is considered an advanced stage of glioma, is associated with a lower overall survival rate than glioma. (Zhang et al., 2019; Pelcovits and Niroula, 2020) LINC00467 knockdown was demonstrated to hinder cell proliferation while promoting cell apoptosis in glioblastoma by sponging miR-339-3p and regulating its target downstream gene, IP6K2. (Liang and Tang, 2020).

### 4.2 Other Functional Mechanisms

Except for functioning as a ceRNA in the underlying mechanisms of various human cancers, LINC00467 can also play different biological roles by interacting with certain proteins, regulating classical signaling pathways, or encoding micro peptides.

#### 4.2.1 Neuroblastoma

Neuroblastoma is a malignant embryonal tumor frequently diagnosed in children that usually occurs in the abdomen and has a high metastatic burden. (Park and Cheung, 2020) The role of LINC00467 in human cancers was first reported by Atmadibrata et al., who identified that LINC00467 transcription was regulated by N-Myc, a type of Myc oncoprotein that is associated with neuroblastoma occurrence. (Atmadibrata et al., 2014) In this study, it was demonstrated that N-Myc could reduce LINC00467 and *RD3* gene promoter activity by directly binding to the Sp1-binding site-enriched region. Through this regulatory mechanism, N-Myc could downregulate the expression of LINC00467 and *RD3*. Moreover, LINC00467 could reduce the mRNA expression of its neighboring protein-coding gene *RD3*. To conclude, N-Myc-mediated suppression of *RD3* mRNA expression could be blocked by N-Myc-mediated suppression of LINC00467. Furthermore, LINC00467 knockdown reduced the survival of neuroblastoma cells by inducing the expression of *DKK1*, a classic tumor suppressor gene. However, in this study, the specific roles of LINC00467 in neuroblastoma cells were not investigated in detail.

#### 4.2.2 Bladder Cancer

Bladder cancer, which mainly occurs in men, is the most common malignancy of the genitourinary system. (Bray et al., 2018) A recent study on the underlying molecular mechanisms of bladder cancer validated that LINC00467 is highly expressed in bladder cancer tissues and cells. LINC00467 was also demonstrated to be a promotion of bladder cancer cell proliferation and invasion. Using the catRAPID algorithm, it was predicted that LINC00467 could bind to NF-kB p65 through several binding sites. Further functional experiments showed that LINC00467 binding to NF-kB p65 increased the stability of this transcription factor and its nuclear translocation, a subcellular process in which NF-kB p65 could be transported into the cell nucleus to regulate gene expression. Moreover, rescue assays demonstrated that LINC00467 could promote the progression of bladder cancer through the NF-κB signaling pathway, which is closely correlated with the occurrence and development of tumors. (Xiao et al., 2021)

#### 4.2.3 LUAD

In LUAD, except for sponging miRNAs, LINC00467 could also exert its function through other molecular mechanisms. A study confirmed that LINC00467 inhibited the expression of HtrA serine peptidase 3 (HTRA3), a downstream gene of LINC00467, by recruiting EZH2 to the HTRA3 promoter, thereby regulating the characteristics of LUAD cells. (Wang et al., 2019) Yang et al. (2019) discovered that STAT1 (signal transducer and activator of transcription 1)-mediated upregulation of LINC00467 promoted LUAD cell proliferation and migration by recruiting enhancer of zeste homolog 2 (EZH2) to downregulate DKK1, which resulted in the inhibition of the Wnt/β-catenin signaling pathway.

#### 4.2.4 NSCLC

In another study, Using the GEPIA and Kaplan–Meier Plotter databases, Zhu et al. found that LINC00467 is highly overexpressed in NSCLC tissues and is associated with advanced clinical stages and poor prognosis. Furthermore, functional experiments confirmed that LINC00467 upregulation was due to TDG (Thymine DNA Glycosylase)-mediated acetylation. Additionally, LINC00467 has been proven to promote NSCLC cell proliferation and metastasis by regulating the Akt signaling pathway. (Zhu et al., 2020).

#### 4.2.5 HCC

LINC00467 can also regulate the initiation and development of HCC through interacting with certain proteins and participating in post-transcriptionally modification. Jiang et al. reported that upregulation of LINC00467 facilitated proliferation and metastasis but hindered cell apoptosis in HCC. A series of functional experiments revealed that the underlying mechanism relied on the direct binding between LINC00467 and insulin-like growth factor-2 messenger RNA-binding protein 3 (IGF2BP3), which increased the stability of the transcript encoding the tumor necrosis factor receptor-associated factor 5 (TRAF5). (Jiang et al., 2020) In addition, LINC00467 can post-transcriptionally reduce the expression of the tumor suppressor NR4A3 by regulating Dicer-dependent RNA splicing, which ultimately contributes to HCC tumorigenesis. (Wang et al., 2020)

#### 4.2.6 CRC

To date, there has only been one article of LINC00467 working by encoding a micro peptide. A study focusing on the role of ATP synthase-associated peptide (ASAP), a micro peptide encoded by LINC00467, reported that ASAP facilitated tumor proliferation through the regulation of mitochondrial ATP production in CRC. Moreover, ASAP expression was negatively correlated with the prognosis of patients with CRC. (Ge et al., 2021).

#### 4.2.7 Testicular Germ Cell Tumors

TGCTs, which can be classified as seminoma or non-seminoma tumors, mainly occur in men aged 20–40 years. In China, its incidence is one every 100,000 people. Furthermore, in China as well as worldwide, the incidence of this disease is gradually increasing (Chia et al., 2010; Greene et al., 2010; Shanmugalingam et al., 2013) Bo et al. reported that LINC00467 can promote TGCT cell migration and invasion by controlling the expression level of AKT3 and affecting the total AKT phosphorylation level. Interestingly, LINC00467 could also inhibit the infiltration of immune cells as well as their activation, indicating that it may be a potential target for anti-PD-1 immunotherapy. (Bo et al., 2021)

#### 4.2.8 GC

In GC, Xu et al. reported that the upregulation of LINC00467 in patients with GC was positively associated with tumor differentiation and the TNM stage. Functional experiments revealed that overexpression of LINC00467 increases GC cell viability and proliferation while suppressing apoptosis by enhancing ITGB3 (integrin subunit beta 3) levels. (Xu et al., 2021)

## 5 Discussion

Recent studies have shown that LINC00467 overexpression is associated with numerous diseases, especially with the progression of various types of tumors. However, the role of LINC00467 in HCC is controversial. In addition, several studies have confirmed that high expression of LINC00467 is associated with poor prognosis in various tumor types. Functional experiments on cancer cells showed that LINC00467 could promote the proliferation, invasion, migration, and inhibition of apoptosis of tumor cells, while silencing LINC00467 exerted the opposite effects. However, owing to the different sample sizes and procedures of each study, the results are non-concluding, and a larger study or multicentre joint studies are needed to further verify these conclusions. Additionally, studying LINC00467 expression levels in human blood or other body fluids would be beneficial for evaluating its clinical application as a promising biomarker for the early diagnosis and prognosis of various diseases. Moreover, silencing LINC00467 could be a suitable therapeutic strategy, but more studies are needed to verify this. *In vivo* studies of LINC00467 with cell-derived and patient-derived xenograft animal models are required to further assess the role of LINC00467 in tumor progression, avoid the limitations of *in vitro* studies, and test new treatment options. However, some of the studies referred to in this paper, such as those conducted for OS, CRC, TGCT, GC, glioma, esophageal cancer, and HNSCC, were performed only *in vitro* without further *in vivo* validation of their findings.

LINC00467 has been demonstrated to function through diverse regulatory mechanisms, mainly acting as a ceRNA of a series of miRNAs, including miR-339, miR-494-3p, miR-125A-3p, miR-4779, miR-7978, miR-20b-5p, miR-107, miR-509-3p, miR-18a-5p, miR-9-5p, miR-217, miR-451A, miR-138-5p, miR-485-5p, miR-299-5p, miR-1285-3p, miR-7-5p, miR-200a, miR-339-3p, and miR-485-5p. By sponging these miRNAs, LINC00467 alters the expression level of downstream genes and influences cell signalling pathways. Central signalling cascades have been found among the pathways regulated by LINC00467; for example, the Wnt/B-catenin pathway in LUAD. However, a few studies failed to fully elucidate the mechanism of action of LINC00467 in some diseases. For example, a study found that LINC00467 could competingly bind with miR-451a, but its downstream gene was not studied. (Bai et al., 2020) Additionally, Bo et al. reported that LINC00467 promotes TGCT cell invasion and migration by activating AKT3. However, the detailed regulatory mechanism between LINC00467 and AKT3 is not illustrated. (Bo et al., 2021) Herein, we expect more comprehensive and in-depth studies to advance the clinical application of LINC00467 in the future.

It is worth noting that studies on HCC have shown that LINC00467 expression is higher in HCC tissues than in controls (Jiang et al., 2020; Wang et al., 2020; Zheng et al., 2020) However, Cai et al. (2019) reported the opposite result in their clinical samples. Furthermore, they found that LINC00467 expression in HCC tissues with metastasis was lower than that in non-metastatic tissues. This contradiction needs to be assessed by further validation with more clinical samples of HCC. Meanwhile, several studies reported that LINC00467 plays a carcinogenic role in HCC by sponging miR-18a-5p or miR-509-3p (Zheng et al., 2020; Li W. et al., 2021), inhibiting NR4A3 (Wang et al., 2020), and binding to IGF2BP3. (Jiang et al., 2020) Cai et al. (2019) studied the LINC00467/miR-9-5p/PPARA axis and concluded that LINC00467 exerts a tumor-suppressive effect in HCC tissues. If the data is real, this contradiction on HCC indicates that LINC00467 plays different roles depending on the cell type.

In addition to regulating the expression level of downstream genes, LINC00467 also functions as a protein-coding RNA, as it encodes several short peptides. Before Andrews and Rothnagel (2014) and Bazzini et al. (2014) reported that ncRNAs could encode hundreds of functional micro peptides, lncRNAs had long been regarded as “transcriptional noise”. Soon afterward, Anderson et al. (2015) found a novel micro peptide myoregulin (MLN), encoded by a lncRNA, plays an important role in regulating skeletal muscle physiology. Similarly, in CRC, LINC00467 can encode a short peptide ASAP, which promotes CRC cell proliferation. Although current studies on LINC00467 and other lncRNAs mainly focus on their interaction with miRNA or proteins, further investigation into the function of these micro peptides encoded by short open reading frames is needed.

Exosomes are a subtype of extracellular vesicles with a diameter of 40–150 nm, secreted by almost all types of living cells (Cocucci and Meldolesi, 2015) An increasing number of studies have confirmed that exosomes are responsible for many biological functions as they transport a large number of functional units, such as DNA, RNA, and proteins. Data from the GEO databases shows that one of the LINC00467 transcripts (NONCODE ID: NONHSAT225914.1) is expressed in exosomes of some samples, such as those from tuberculosis patient serum and HepG2s (Hepatocellular Carcinoma Cell Line Exosomes). Further studies should be conducted to determine the role of exosome-mediated transport of LINC00467 in various diseases.

In this review, the latest research progress on LINC00467 is summarized, and its biological mechanisms and clinical application value in various tumors are detailed. The described studies highlight that LINC00467 plays a remarkable role as an oncogene. Further research on LINC00467 and its mechanism of action may contribute to its use for disease diagnosis, targeted therapy, and prognostic evaluation in the future.
